# Optical Genome Mapping versus Whole-Genome Sequencing in the Clinical Diagnosis of Gynecologic Mesenchymal Tumors

**DOI:** 10.1016/j.jmoldx.2025.11.003

**Published:** 2025-11-29

**Authors:** Karin Wallander, Yingbo Lin, Vadym Ivanchuk, Valeria Difilippo, Venkatesh Chellappa, Sarath K. Murugan, Ingegerd Öfverholm, Robert Bränström, Karolin H. Nord, Joseph Carlson, Felix Haglund de Flon

**Affiliations:** ∗ Department of Oncology-Pathology, Karolinska Institutet, Stockholm, Sweden; † Clinical Genetics and Genomics, Karolinska University Hospital, Stockholm, Sweden; ‡ Clinical Pathology and Cancer Diagnostics, Karolinska University Hospital, Stockholm, Sweden; § Department of Medical Epidemiology and Biostatistics, Karolinska Institutet, Stockholm, Sweden; ¶ Division of Clinical Genetics, Department of Laboratory Medicine, Lund University, Lund, Sweden; || Department of Molecular Medicine and Surgery, Karolinska Institutet, Stockholm, Sweden; ∗∗ Department of Breast Cancer, Endocrine Tumors and Sarcoma, Karolinska University Hospital, Stockholm, Sweden; †† Department of Pathology, City of Hope, Duarte, California

## Abstract

Optical genome mapping (OGM) enables high-resolution detection of structural variants (SVs) and copy number aberrations (CNAs) using ultralong DNA molecules and minimal bioinformatics processing. Its diagnostic utility in solid tumors remains underexplored. Whole-genome sequencing (WGS) offers comprehensive variant detection but is resource intensive. This study presents a technical benchmarking of OGM versus WGS for mesenchymal tumors of the gynecologic tract. Twenty-five uterine mesenchymal tumors were prospectively analyzed using matched WGS, transcriptome sequencing, and OGM. Detected SVs, CNAs, and fusion genes were compared across platforms. OGM identified structural driver events in 80% of cases and demonstrated high concordance with WGS for major CNAs and translocations. In select cases, OGM resolved complex rearrangements not clearly defined by WGS, including a *PLAG1*::*RERE* fusion and an embedded inversion in a *RAD51B*::*HMGA2* event. Conversely, WGS uniquely detected a truncating *NF1* translocation and a *TSC2*::*SENP3* fusion, both clinically significant. OGM is a technically robust platform for SV and CNA detection in mesenchymal tumors, and it may serve as an efficient alternative to sequencing-based cytogenomic approaches in selected clinical contexts, especially in tumors known to be driven by gross chromosomal rearrangements. WGS provides a comprehensive view of the cancer genome, suitable for tumors driven by single-nucleotide variants, SVs, and CNAs. The choice between platforms should be guided by clinical context, diagnostic needs, and available resources.

Mesenchymal tumors of the female gynecologic tract comprise a diverse and clinically significant group of neoplasms that may present with abnormal uterine bleeding, pelvic pain, infertility, and, albeit rarely, malignant transformation. Among these, uterine leiomyomas (benign smooth muscle tumors) are the most prevalent, affecting most of adult women. They represent a leading cause of gynecologic morbidity.^1^ Although histologically benign, leiomyomas frequently require surgical intervention, posing substantial health care and psychosocial burdens.

From a genomic perspective, leiomyomas typically exhibit limited genomic complexity, most commonly characterized by *MED12* single-nucleotide variants (SNVs) or *HMGA2* rearrangements. Less frequently, biallelic loss of *FH* or deletions in *COL4A5* and *COL4A6* are observed, often in association with hereditary syndromes, such as hereditary leiomyomatosis and renal cell carcinoma, or Alport syndrome.^2^, ^3^, ^4^, ^5^, ^6^ In contrast, leiomyosarcomas (LMSs) are highly aggressive malignancies distinguished by complex karyotypic abnormalities and frequent mutations in *TP53*, *RB1*, *ATRX*, and occasionally *PTEN*.^7^ Morphologically, they display marked cytologic atypia, elevated mitotic activity, and coagulative tumor necrosis.^8^

The current World Health Organization Classification of Tumors recognizes at least 27 distinct mesenchymal tumor entities within the gynecologic tract, many of which are defined by unique molecular alterations.^8^ Although histopathologic examination remains the diagnostic gold standard, tumors with overlapping features or borderline morphology present significant diagnostic challenges. In such cases, molecular profiling has proven indispensable for refining diagnoses and informing therapeutic strategies.^9^ Consequently, the integration of molecular diagnostics into routine clinical workflows is becoming increasingly common.

Despite advances in next-generation sequencing, structural variants (SVs), such as cryptic rearrangements, complex translocations, and chromoanagenesis events, remain difficult to resolve. Optical genome mapping (OGM) is a novel, high-resolution platform capable of visualizing ultralong DNA molecules to detect large insertions, deletions, inversions, and translocations at high resolution. In a recent study, Ghabrial et al^10^ demonstrated the utility of OGM in a cohort of 60 bone and soft tissue tumors, identifying structural variants and copy number alterations in 91% of cases with known aberrations, and revealing complex genomic events, including chromoanagenesis, that were not detected by conventional cytogenetic methods. However, OGM has not yet been systematically compared with whole-genome sequencing (WGS).

The Karolinska University Hospital has adopted a comprehensive genomic diagnostic approach that includes WGS and whole-transcriptome sequencing (WTS), and DNA methylation profiling for tumors suspected to be sarcomas.^11^^,^^12^ This study evaluates the feasibility and diagnostic value of OGM as an adjunct or replacement to WGS and WTS by analyzing a cohort of 25 uterine mesenchymal tumors. Although cytogenomics is not the main diagnostic determinant for this tumor entity, gynecologic sarcomas constitute a relatively uncharacterized group that benefit from a comprehensive genomic description. The objective is to determine whether OGM enhances genomic resolution and contributes to a more accurate classification of mesenchymal neoplasms in the gynecologic tract.

## Materials and Methods

### Patient Inclusion

This study included 25 patients with gynecologic tumors that were clinically suspected to be sarcomas. Suspicion of malignancy was determined at the preoperative multidisciplinary conference by the treating clinician, based on imaging, medical history, and physical examination. A preoperative biopsy was rarely performed. All cases had previously undergone comprehensive WGS and WTS at the institution.^11^ Inclusion criteria were the presence of a gynecologic tumor with a preoperative suspicion of sarcoma and the availability of fresh frozen tumor tissue. All surgical procedures were performed at the Sarcoma Center, Karolinska University Hospital (Stockholm, Sweden). The Karolinska Hospital is a tertiary center for gynecologic malignancies and sarcomas, with a patient uptake from approximately a third of the country. Frozen tumor samples were used for genomic DNA extraction and subsequent OGM.

### Ethics

The study was designed in accordance with Swedish law and the Declaration of Helsinki. The study was approved by the Swedish Ethical Review Authority (identifiers 2022-05409-01 and 2013 1979-31, with amendment 2018/2124-32). All participants gave written informed consent before enrollment in the study.

### Histopathologic Assessment

All histologic tumor samples were prepared according to clinical guidelines, and the diagnoses were established by subspecialized, board-certified pathologists at the Karolinska University Hospital Pathology and Cancer Diagnostics institution, following World Health Organization criteria.

### Sequencing and Variant Analysis

Genomic DNA and total RNA were extracted from fresh frozen tumor tissue, while matched peripheral blood samples or normal tissue served as germline controls. Sampling for DNA extraction was performed by an experienced tumor pathologist, ensuring high tumor tissue content. Library preparation, high-throughput sequencing, and primary data processing were performed as previously described.^11^ Tumor samples were sequenced at a target depth of 100×, and germline controls at 30×, using Illumina (San Diego, CA) sequencing platforms. A total of 800 ng of tumor RNA from each case was submitted for whole transcription sequencing. PolyA enrichment, fragmentation, and library preparation were done using the Illumina Stranded mRNA Prep kit, following the manufacturer’s instructions. Paired-end 150-bp sequencings were performed on the NovaSeq 6000 platform (Illumina). The generated data were processed with the RNAfusion pipeline (*https://zenodo.org/records/3946477*, last accessed June 4, 2025).

DNA variant calling and annotation were conducted using the in-house bioinformatics pipelines BALSAMIC (*https://balsamic.readthedocs.io/en/v12.0.2*, last accessed December 22, 2023) and AutoSeq,^13^ which integrate multiple SV detection algorithms: Manta version 1.6.0 [Research Resource Identifier (RRID): SCR_022997], Delly version 1.0.3 (RRID: SCR_004603), and TIDDIT version 3.3.2 (RRID: SCR_024361). Copy number aberrations (CNAs) were assessed using both tumor-normal and tumor-only approaches: ascatNgs version 4.5.0 for tumor-normal analyses; and Delly version 1.0.3 and CNVpytor version 1.2.1 (RRID: SCR_021627) for tumor-only analyses.^13^ Aberrant cell fraction estimations from ascatNgs were cited as tumor purities for each case.

Somatic SNVs were filtered and curated following institutional standard operating procedures.^14^ Structural variants were prioritized on the basis of read support and the known or potential relevance of the affected genes. All candidate SVs were manually validated using the Integrative Genomics Viewer (*https://igv.org*).^15^

CNAs were assessed at both chromosomal and gene levels. Analyses included the identification of broad chromosomal gains and losses, as well as focal amplifications and homozygous deletions affecting cancer-associated genes. Arbitrarily, a CNA profile was considered complex if it exhibited more than five large-scale (megabases) chromosomal aberrations, focal amplifications or deletions, or evidence of chromoanagenesis (operationally defined as the presence of ≥10 SVs and/or CNAs clustered within a single chromosomal region).

The genomic complexity score was calculated to assess the genome-wide complexity for each case. This score operates under the premise that low genomic complexity is characterized by a limited number of copy number alterations, whereas high genomic complexity corresponds to widespread copy number alterations across the genome. The computational method used to derive the scores followed the approach previously described by Difilippo et al.^16^ As genomic complexity is a relative measure, there is no defined threshold distinguishing low from high complexity. An expanded cohort of 41 gynecologic mesenchymal tumor cases were used for comparisons, to increase the number of cases and thereby the chance of detecting tendencies. All samples were extracted from the same inclusion procedure as the 25 cases presented in this study, and all cases were processed the same way. Genomic complexity scores were calculated on the basis of the outputs from allele-specific copy number analysis of tumors, except for eight, which were based on CNVkit. Genomic complexity scores were plotted against patient age and stratified by the mutational status of *TP53*, *RB1*, and *ATRX*, enabling exploration of potential associations between genomic complexity and molecular alterations.

### Transcriptomic Analysis

RNA-sequencing data were processed using the nf-core/rnafusion pipeline version 2.3.4 (*https://zenodo.org/records/3946477*, last accessed June 4, 2025), which incorporates multiple fusion detection tools, including FusionCatcher,^17^ Arriba,^18^ and STAR-Fusion.^19^ This multicaller approach was used to increase sensitivity and specificity in the identification of gene fusion events, a critical diagnostic feature in mesenchymal tumors.

### Optical Genome Mapping

Sampling was performed by an experienced tumor pathologist (F.H.d.F.). Fresh frozen tumor tissue was sectioned at a thickness of 20 μm and processed using the Bionano Prep SP Tissue and Tumor protocol (Bionano Genomics, San Diego, CA). Homogenization was performed on ice to preserve DNA integrity, followed by centrifugation and serial washing of the tissue pellet. High-molecular-weight genomic DNA was extracted using Nanobind magnetic disks and subjected to gentle mixing on a HulaMixer (Thermo Fisher Scientific, Waltham, MA). DNA concentration was quantified with the Qubit dsDNA BR Assay Kit (Thermo Fisher Scientific).

Labeling of genomic DNA was performed using the Bionano Prep Direct Label and Stain protocol. Labeled DNA was loaded onto Saphyr Chips and analyzed using the Saphyr Optical Genome Mapping System (Bionano Genomics). Quality control metrics adhered to standard thresholds: a minimum DNA molecule length of ≥150 kbp, N50 of ≥150 kbp, and a label density of ≥15 labels per 100 kbp.

Variant detection was performed using the Rare Variant Analysis pipeline in Bionano Access version 1.8. Filtering criteria were based on guidelines from an international OGM working group for hematologic malignancies,^20^ and included the following:

• SV masking filter: all structural variants
• Population frequency filter: retained variants present in ≤1% of the control database (matched for labeling enzyme)
• CNA masking filter: all copy number variants

CNAs were visualized in a whole genome view, allowing the identification of both broad chromosomal imbalances and focal alterations, such as amplifications or homozygous deletions. Chromoanagenesis was, as specified also for the sequencing approach, defined as ≥10 SVs and/or CNAs within a single chromosomal region.

To assess concordance between OGM and WGS, a two-way comparison was performed, focusing on focal deletions and amplifications of clinically relevant genes, translocations resulting in tumor suppressor gene truncations, and rearrangements with the potential to generate fusion genes. Variant calls were independently derived from OGM and WGS data sets and subsequently cross-validated by manual inspection in the alternate data set. Fraction of genome altered (FGA) was calculated from WGS and OGM data (*https://github.com/ki-sarcoma/wgs-vs-ogm/blob/main/FGA/methods.md*, last accessed October 20, 2025), giving an approximation of CNA prevalence within the samples. CNA segments from autosomes (chromosomes 1 to 22) with a minimum size of 50 kbp and log2 ratio of ±0.2 were included in the calculations, and adjustment for purity was performed.

## Results

### Tumor Characteristics

A total of 25 patients were included: 12 with conventional LMSs, 11 with leiomyomas, one with a perivascular epithelioid cell tumor (PEComa), and one with a uterine lipoblastoma. Among the LMS cases, eight were sampled from primary tumors and four from metastatic sites. The mean age across the cohort was 56 years (range, 39 to 82 years). The mean tumor size for LMS and PEComa was 12 cm (range, 0.5 to 27 cm). The leiomyomas, initially suspected preoperatively to be sarcomas, also had a mean size of 12 cm (range, 6 to 21 cm). All samples had a tumor purity >20%, and the median tumor purity in the cohort was 86%.

### Tumor Genomics by Whole-Genome Sequencing

**Figure 1:**
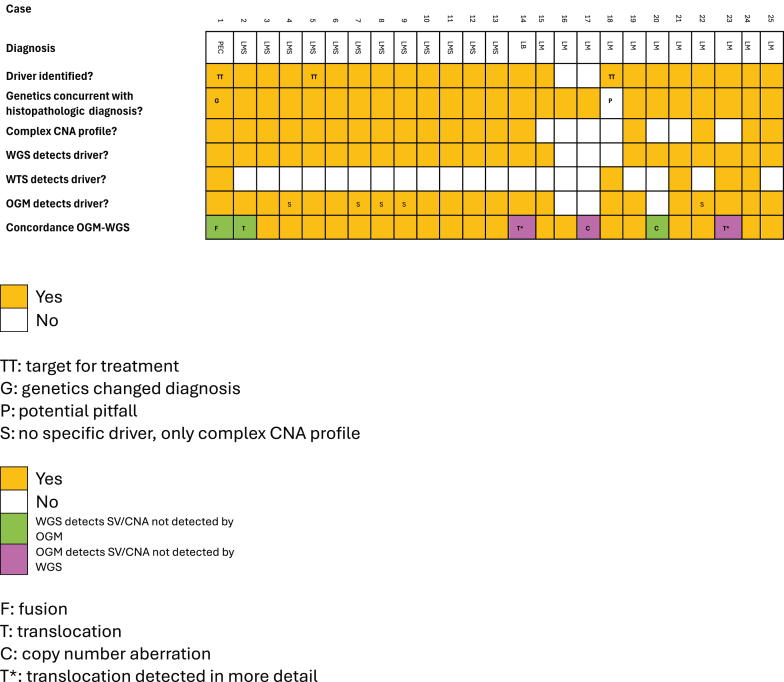
Oncoplot. Diagnoses and summary of genomic findings for each study participant. *n* = 25. CNA, copy number aberration; LB, lipoblastoma; LM, leiomyoma; LMS, leiomyosarcoma; OGM, optical genome mapping; PEC, perivascular epithelioid cell; SV, structural variant; WGS, whole-genome sequencing; WTS, whole-transcriptome sequencing.

A summary of the genomic alterations is presented in Table 1 and visualized in Figure 1. WGS identified somatic driver events, defined as key genomic findings, in 23 of 25 cases.

**Table 1:** Clinically Relevant Genomic Findings for Each Case (*n* = 25), Based on WGS, WTS, and OGM

Case	Diagnosis	Key genomic finding	GCS	CNAs
PEC-1	PEComa	*TSC2*, *ATRX* (homdel), *RB1* (homdel), *TSC2*::*SENP3*∗	0.19	Complex
LMS-2	Leiomyosarcoma	*TP53*, *TP53* (homdel), *ATRX*, *RB1* (trunc), *NF1* (trunc)∗	0.63†	Complex
LMS-3	Leiomyosarcoma	*TP53*, *ATRX*, *RB1* (trunc)	0.55†	Complex
LMS-4	Leiomyosarcoma	*TP53*, *ATRX*, *RB1*	0.78†	Complex‡
LMS-5	Leiomyosarcoma§	*CDKN2A* (trunc), tumor mutational burden	0.38†	Complex
LMS-6	Leiomyosarcoma	*CDKN2A/B* (homdel)	0.44	Complex
LMS-7	Leiomyosarcoma	*TP53*, *RB1*	0.43	Complex
LMS-8	Leiomyosarcoma§	*TP53*, *ATRX*	0.66	Complex
LMS-9	Leiomyosarcoma§	*TP53*	0.46	Complex‡
LMS-10	Leiomyosarcoma	*ATM*, *CDKN2A/B/C* (homdel)	0.43	Complex
LMS-11	Leiomyosarcoma	*TP53* (homdel), *ATRX*del, *RB1* (homdel)	0.56	Complex
LMS-12	Leiomyosarcoma	*CDKN2C* (homdel)	0.37	Complex‡
LMS-13	Leiomyosarcoma§	*TP53*, *PTEN* (homdel), *ATRX* (trunc)	0.62	Complex
LB-14	Lipoblastoma	*PLAG1*::*RERE* (three-way translocation)¶	0.24	Complex
LM-15	Leiomyoma	*FGFR1*, *RAD51B* (trunc)	0.2†	X del
LM-16	Leiomyoma	No	0.13†	1p, 15, 19q del
LM-17	Leiomyoma	No	0.03	1q del¶
LM-18	Leiomyoma	*FGFR1*::*TACC1*	0.12	1q, 10cen del
LM-19	Leiomyoma	*RAD51B* (trunc)	0.25	Complex
LM-20	Leiomyoma	*MED12*	0.04	13q del∗
LM-21	Leiomyoma	*COL4A5*::*RAD51B*	0.02	No
LM-22	Leiomyoma	*HRAS*	0.11	Complex
LM-23	Leiomyoma	*RAD51B*::*HMGA2* (inversion within translocation)¶	0.03	No
LM-24	Leiomyoma	*RAD51B*::*MSRB3*	0.18	Complex
LM-25	Leiomyoma	*HMGA2* rearrangement	0.09	Complex

All leiomyosarcomas are conventional. When discrepancies exist between detection methods, they are specified with superscript according to table legend.

CNA, copy number aberration; del, deletion; GCS, genomic complexity score; homdel, homozygous deletion with copy number state close to 0; OGM, optical genome mapping; PEComa, perivascular epithelioid cell tumor; trunc, truncation (translocation interrupting the gene); WGS, whole-genome sequencing; WTS, whole-transcriptome sequencing.

∗ Structural variant/CNA only detected by WGS.

† On the basis of CNVkit instead of allele-specific copy number analysis of tumors.

‡ B-allele frequency identifies large regions of copy-neutral loss of heterozygosity.

§ Metastatic sample.

¶ Structural variant/CNA only detected by OGM.

**Figure 2:**
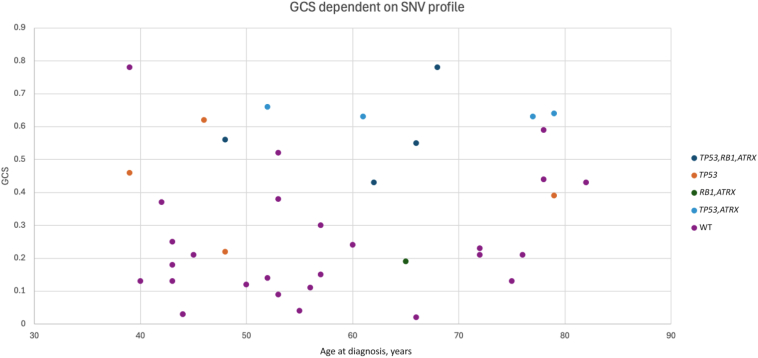
Genomic complexity score (GCS). Genomic complexity score plotted against age at diagnosis, for an expanded gynecologic mesenchymal tumor cohort, including all cases from the present study. Each case is represented by a dot, colored on the basis of genomic status according to the figure legend on the right. Cases presenting with pathogenic either single-nucleotide variants (SNVs) or structural variants in the genes *TP53*, *ATRX*, and *RB1* in whole-genome sequencing have different color codes depending on the combination of variants. *n* = 41. WT, wild type.

Leiomyomas (*n* = 11) exhibited a limited but heterogeneous set of genomic alterations. SVs were detected involving *RAD51B* (*n* = 5), *HMGA2* (*n* = 2), and *FGFR1* (*n* = 1), whereas likely pathogenic SNVs were identified in *MED12* [NM_005120, c.130G>A, p.(Gly44Ser); *n* = 1], and *HRAS* [NM_17695, c.183G>C, p.(Gln61His); *n* = 1], both accessible in National Center for Biotechnology Information Reference Sequence Database (RefSeq, *https://www.ncbi.nlm.nih.gov/refseq*, last accessed October 20, 2025). All alterations were mutually exclusive. Two leiomyomas lacked identifiable driver events: one exhibited isolated deletions involving chromosomes 1p, 15pq, and 19q, whereas the other displayed a diploid genome without significant copy number aberrations. Overall, CNAs were few in leiomyomas, with recurrent alterations affecting chromosome 1. Chromosomal complexity, defined as the presence of more than five CNAs, was observed in four cases. Notably, four leiomyomas and the lipoblastoma displayed a complex CNA profile as per our definition, but the genomic complexity scores were still lower than all but one malignant case. The mean genomic complexity score in the benign group was 0.12, compared with 0.50 in the malignant group (Table 1 and Figure 2).

Leiomyosarcomas (*n* = 12) demonstrated widespread chromosomal instability, with high levels of genomic complexity. All cases harbored multiple driver alterations, including inactivation of *TP53* (*n* = 8), *ATRX* (*n* = 7), *RB1* (*n* = 6), and *CDKN2A* (*n* = 3). Additional findings included high tumor mutational burden in one case and *NF1* inactivation in another. The single PEComa case showed high-grade sarcomatous morphology, with whole-genome and transcriptome sequencing revealing extensive copy number alterations and biallelic inactivation of *TSC2*.

To our knowledge, the uterine lipoblastoma (LB-14) represented the first reported case of its kind. Genomic analysis revealed a novel *PLAG1*::*RERE* fusion and a diploid genome with moderate structural complexity.

### Tumor Genomics by Optical Genome Mapping

OGM identified either clinically relevant focal driver events involving known tumor suppressor genes or oncogenes, or a complex CNA profile, in 22 of 25 cases (88%) (Table 1 and Supplemental Table S1).

Chromoanagenesis, defined as a localized cluster of complex rearrangements and CNAs, was observed in 12 of 25 cases (48%), with the most frequently affected chromosomes being 1 (*n* = 3), 2 (*n* = 3), 4 (*n* = 2), 6 (*n* = 2), 12 (*n* = 2), and 13 (*n* = 2) (Supplemental Table S1). Notably, all but three benign cases lacked evidence of chromoanagenesis.

In the leiomyoma group, recurrent involvement of *RAD51B* was observed, including three fusion events and two truncating alterations. Among the leiomyosarcomas, OGM revealed recurrent truncating events affecting *ATRX*, *TP53*, *RB1*, *CDKN2A*, *CDKN2B*, and *CDKN2C*, mediated by either focal deletions or gene-disrupting translocations.

Translocations (involving at least two different chromosomes) were detected in all but three leiomyomas (Supplemental Table S1). Although most benign tumors exhibited only one or a few such events, leiomyosarcomas showed widespread chromosomal involvement, often accompanied by chromoanagenesis patterns, consistent with their high genomic complexity.

### Comparison between Whole-Genome Sequencing and Optical Genome Mapping

A high degree of concordance was observed between WGS and OGM in the detection of both SVs and CNAs, as summarized in Table 1.

CNA profiles were compared across both platforms using chromosomal-level visualizations of large-scale gains and losses, focal amplifications, and homozygous deletions. Discrepancies were minimal and limited to two leiomyoma cases: a deletion on chromosome 1q was detected by OGM but missed by WGS, whereas a deletion on chromosome 13q was identified by WGS but not detected by OGM. Three leiomyosarcomas exhibited highly complex CNA profiles that posed challenges for ploidy determination (Supplemental Figure S1). These cases exhibited widespread chromosomal imbalances, with large-scale genomic gains and losses affecting most chromosomes. Although relative gene dosage levels were consistent between WGS and OGM, the B-allele frequency plots generated from WGS data revealed regions of copy-neutral loss of heterozygosity, a feature not detectable by the currently used OGM analysis software.

**Figure 3:**
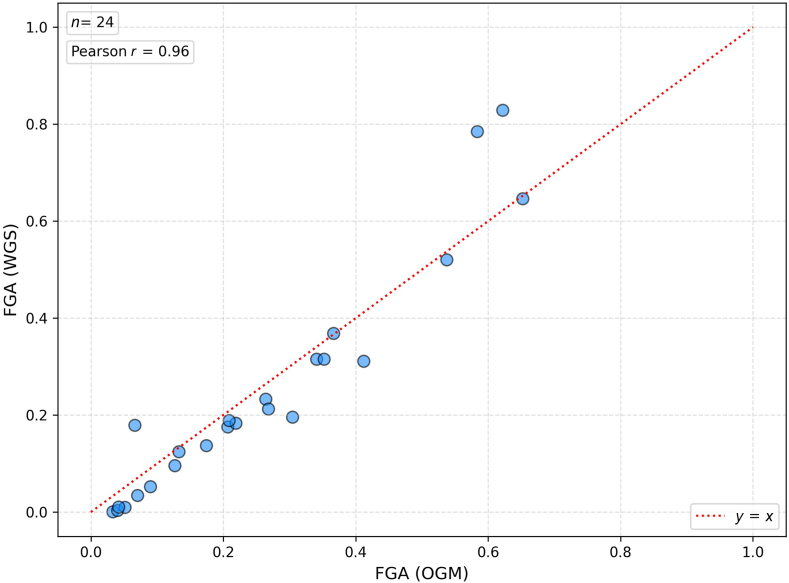
Fraction of genome altered (FGA). Comparison of FGA calculated from whole-genome sequencing (WGS; *y* axis) and optical genome mapping (OGM; *x* axis) data sets (case LM-21 was excluded because of missing data). *n* = 24.

There was a high level of concordance between FGA based on WGS and OGM, as can be seen in Figure 3. As can be seen in Supplemental Figure S2, FGA and genomic complexity score also correlated well.

**Figure 4:**
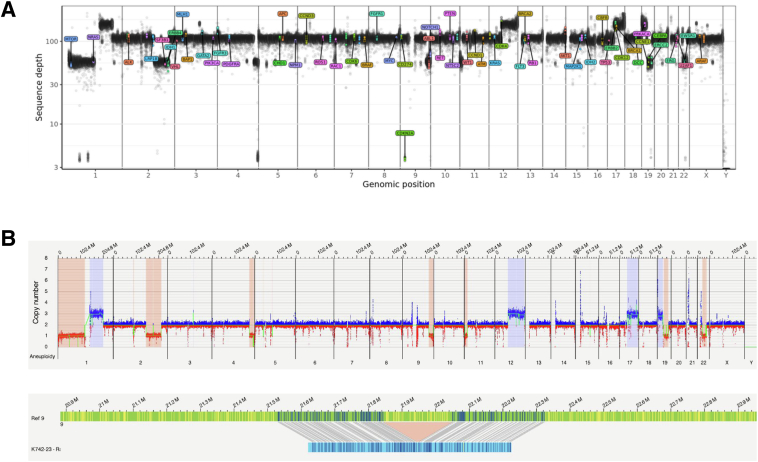
Copy number aberration (CNA) profiles. Whole-genome view visualizations of large or extreme amplitude CNAs. **A:** CNA profile for a leiomyosarcoma (case LMS-10), based on whole-genome sequencing. AutoSeq output of gene dose (*y* axis) for each chromosome (*x* axis). Genes relevant for cancer have colored label tags on each genomic position. A focal deletion of *CDKN2A* is visible on chromosome 9. **B:** CNA profile for a leiomyosarcoma (case LMS-10), based on optical genome mapping. **Top panel:** Bionano access whole-genome view, with gene dose on the *y* axis and chromosome position on the *x* axis. **Bottom panel:** The *CDKN2A* deletion is not evident in the whole-genome view, but it is presented in the genome browser. The local consensus map (blue bar) maps to chromosome 9 [green reference (Ref) bar] except for the deleted region in the middle. M, million.

CNA profile visualizations from variant annotation tools are compared in Figure 4. For instance, a deletion involving *CDKN2A* was visible in the WGS whole-genome overview but was not readily apparent in the OGM whole-genome display. Nevertheless, because of OGM's filtering pipeline, which includes truncating structural variants in known tumor suppressors, the *CDKN2A* deletion was correctly identified and annotated as an SV in the OGM output.

Concordance in SV detection was similarly high. All but one truncating variant in tumor suppressor genes, and all but one fusion gene, were concordantly identified by both WGS and OGM. The two discordant events, detected by WGS but not by OGM, included a truncating translocation affecting *NF1* and a *TSC2*::*SENP3* fusion, the latter confirmed by WTS.

**Figure 5:**
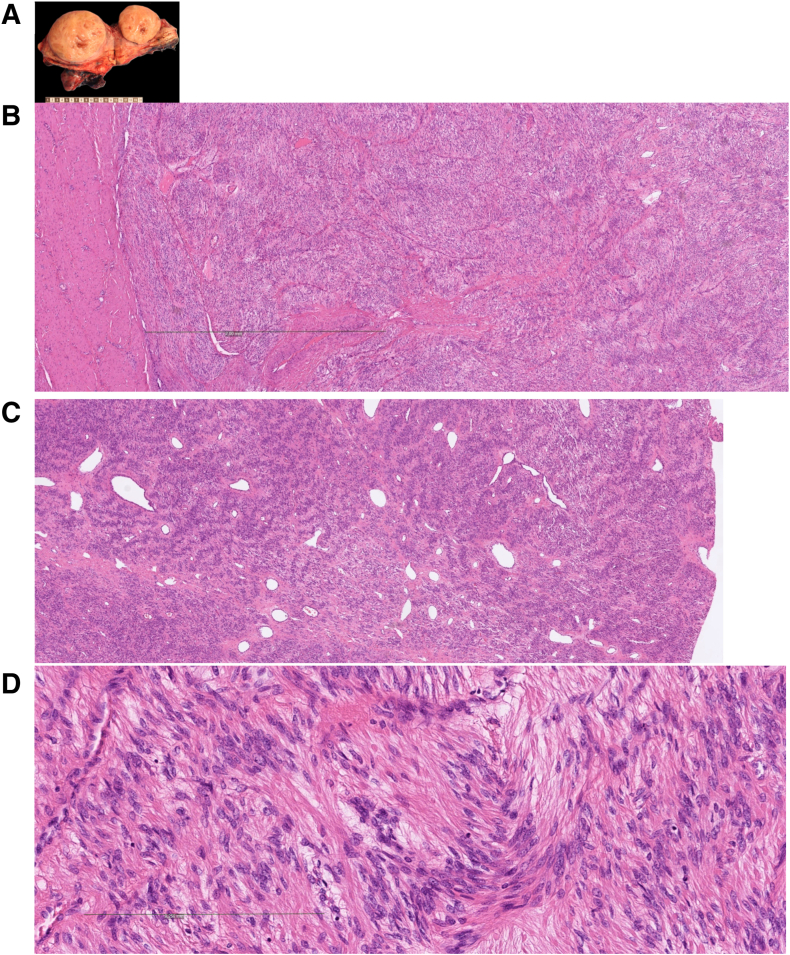
Histologic image of cellular leiomyoma (LM-18) with an *FGFR1::TACC1* fusion. **A:** Submucosal leiomyoma of the uterus, measuring 11 × 9.5 × 8.0 cm. The lesion is yellow-tan with a trabeculated, whorled cut surface with a rubbery consistency. Foci of degradation with cystic change are present, but no hemorrhage or necrosis was identified. The external surface is smooth without evidence of invasion into adjacent myometrium. **B**–**D:** Histology of low (**B** and **C**) and high power (**D**) shows a spindle cell neoplasm composed of intersecting fascicles of uniform smooth muscle cells with elongated, blunt-ended nuclei and inconspicuous nucleoli. The tumor cells demonstrate extensive areas of nuclear palisading, imparting a focal Verocay-like pattern. Cytoplasm is eosinophilic and fibrillary. Mitotic activity is low (≤2/10 high-power fields), and there is no tumor cell necrosis or significant cytologic atypia. **B:** Thin-walled blood vessels are present, and no infiltrative growth into surrounding myometrium is observed. Scale bars: 2 mm (**B**); 0.25 mm (**D**). Original magnification, ×40 (**C**).

WTS further identified an *FGFR1*::*TACC1* fusion in a leiomyoma (LM-18) (Figure 5) that was not detected by either WGS or OGM. This is a known fusion between two genes situated 260 kilobases apart in the 8p11.22-23 region. In this case, OGM also failed to detect any key structural variants, although both platforms showed concordant findings for a 1q deletion and a deletion involving the centromeric region of chromosome 10.

Importantly, OGM provided enhanced resolution in characterizing complex rearrangements in two cases. In the uterine lipoblastoma (LB-14), OGM resolved a three-way translocation underlying a *PLAG1*::*RERE* fusion, whereas in a leiomyoma with a *RAD51B*::*HMGA2* fusion (LM-23), OGM revealed an inversion embedded within the translocation, a detail not resolved by WGS alone. These cases exemplified the added structural detail achievable with OGM, particularly for complex or multistep rearrangements.

### Clinical Relevance of Genomic Profiling

In 24 of 25 cases, the genomic profile was concordant with the histopathologic diagnosis. One leiomyoma (LM-18) presented a finding that could pose potential diagnostic challenges. It harbored an *FGFR1*::*TACC1* fusion, an alteration previously described in a case of undifferentiated spindle cell sarcoma. Although histologically benign, the presence of this fusion gene raised concern about possible misclassification.

Two leiomyomas (LM-16 and LM-17) lacked identifiable driver events (Figure 1), a finding compatible with a benign classification, but not diagnostic of leiomyoma specifically. Conversely, in one case, the genomic findings led to a revision of the initial diagnosis (PEC-1). Originally classified as an epithelioid leiomyosarcoma, the tumor was found to have biallelic *TSC2* inactivation. This prompted histologic re-evaluation and immunohistochemical confirmation, resulting in reclassification as a PEComa (Table 1 and Figure 1).

Several alterations identified across the cohort may hold therapeutic significance. These included *TSC2* inactivation (resulting in potential sensitivity to mammalian target of rapamycin inhibitors), the *FGFR1* fusion [a candidate for fibroblast growth factor receptor (FGFR)–targeted therapies], and a case of high tumor mutational burden in a leiomyosarcoma (which could support the use of immune checkpoint inhibitors according to the American College of Molecular Genetics/Association for Molecular Pathology) or European Society for Medical Oncology Scale for Clinical Actionability of Molecular Targets criteria).

## Discussion

This prospective descriptive study systematically compared WGS and OGM in detecting clinically relevant genomic alterations in gynecologic mesenchymal tumors, and evaluated the clinical utility of genomic profiling in this cohort. The findings demonstrate that WGS offers the most comprehensive genomic profiling, capturing SNVs, fusion genes, and detailed CNA profiles. OGM, by contrast, provides high-resolution SV detection and whole-genome visualization with minimal computational demands.

To date, most clinical evaluations of OGM have focused on hematologic malignancies, using karyotyping or fluorescence *in situ* hybridization as benchmarks. OGM has proven to be a valuable alternative or complement to these conventional cytogenetic methods, because of its relatively straightforward laboratory protocols and reduced bioinformatics burden. Unlike WGS, OGM provides an intuitive visual map of large SVs, making it appealing in settings with limited bioinformatics infrastructure.

This is the first study to clinically compare WGS and OGM for solid tumors, to our knowledge. Ghabrial et al^10^ evaluated OGM in 60 mesenchymal tumors, primarily benchmarking against cytogenetics, while also incorporating next-generation sequencing for SNV detection. Their work emphasized the clinical utility of OGM when filtered with browser extensible data (BED) files targeting cancer-relevant genes.^10^ Similarly, Baelen et al^21^ applied OGM to 38 mesenchymal tumors and identified 91% of known driver variants previously seen in karyotypes, along with complex rearrangements, such as chromoplexy.

Although molecular stratification of leiomyosarcoma is not yet standard clinical practice, Dermawan et al^22^ proposed a genomic risk model based on *TP53*, *ATRX*, *RB1*, and 12q deletions. Unlike in hematologic oncology, where genome ploidy and structural complexity directly inform diagnosis and prognosis,^23^^,^^24^ such metrics are not routinely used in leiomyosarcoma management. Although most CNA profiles were concordant between platforms, copy-neutral loss of heterozygosity regions detectable by WGS were not consistently identified by OGM because of limitations in B-allele frequency visualization within the Bionano Access interface. Although alternative algorithms exist in the VIA software version 7.1 (Bionano Genomics), copy-neutral loss of heterozygosity remains a known limitation in rare variant assembly pipelines,^25^ and certain low hypodiploid states may also be missed by OGM.^26^

In the cohort, two clinically significant translocations, one truncating *NF1* and another forming a *TSC2*::*SENP3* fusion, were detected by WGS but missed by OGM. The latter was validated through WTS. Although OGM provides a resolution of approximately one label per 5 kbp (targeting CTTAAG motifs), certain genomic regions may lack motif coverage, potentially impairing detection sensitivity. Conversely, OGM more precisely delineated two complex SVs: a three-way translocation forming *PLAG1*::*RERE* in lipoblastoma, and an inversion embedded in a *RAD51B*::*HMGA2* fusion in a leiomyoma. These findings align with prior reports demonstrating OGM's superiority over cytogenetics in mapping cryptic SVs and CNAs, and its potential added value to WGS and WTS.^27^^,^^28^

As expected, OGM does not detect SNVs. Likely pathogenic SNVs were identified in 10 of 13 malignant cases (77%) and 5 of 12 benign cases (42%). All of these cases also exhibited either a complex CNA profile or a structural driver event. SNV detection remains crucial for identifying therapeutically actionable alterations. Although no US Food and Drug Administration–approved therapies currently exist specifically for leiomyosarcomas, the *FGFR1* fusion in case LM-18 could represent a target for pazopanib,^29^ and the high tumor mutational burden observed in case LMS-5 could support the use of immune checkpoint inhibitors.^30^ Although *CDKN2A* alterations are frequent, they are not currently targetable, although palbociclib has shown potential benefit in some uterine sarcomas.^31^

Additionally, genomic complexity score derived from WGS may offer prognostic value. It has been shown to accurately reflect tumor complexity in osteosarcoma.^16^^,^^32^ SNVs in *ATRX*, *TP53*, and *RB1*, detected in six leiomyosarcomas, are known adverse markers. The co-occurrence of *ATRX* and *RB1* alterations has been associated with high-risk molecular subtypes.^32^ In the data set, higher genomic complexity score was frequently observed in such double-mutant tumors (Figure 2). Although normal cell contamination can affect genomic complexity score quantification, no such confounding was verified in this cohort. In Supplemental Figure S2, FGA and genomic complexity score in general show similar results, with higher values in the malignant cases. Chromoanagenesis might, in theory, increase the complexity score relatively more than FGA. That tendency was not clear in the small cohort. High mutational burden is uncommon in mesenchymal tumors, and the leiomyosarcoma case LMS-5 also showed a relatively high FGA and genomic complexity score. On the basis of the genomic findings, the histology was carefully reviewed, with sustained diagnosis. LMS-5 had received chemotherapy before sampling, which could be a plausible explanation for the elevated mutational burden.

Although both WGS and OGM detect major structural alterations, the filtration of clinically meaningful variants varies substantially. Reliable interpretation depends on curated gene panels and established pipelines, particularly in mesenchymal tumors, where the significance of some aberrations remains uncertain. Analogous to hematologic oncology, CNA prevalence (such as FGA) and overall genomic complexity, irrespective of specific gene involvement, may emerge as prognostically significant markers.^32^

The major restriction in this study is the current narrow diagnostic utility of cytogenetics for this specific diagnostic group. OGM is mainly considered a replacement for karyotyping and other cytogenetic approaches. Given that WGS would capture most significant genomic aberrations (SNVs and CNAs) diagnostic for leiomyomas and leiomyosarcomas,^8^ the added value of karyotyping or OGM is limited in standard of care. This study, therefore, focuses on technical comparisons between the methods, as the clinical benefit is expected to favor WGS, which captures SNVs as well as CNAs and SVs. Also, this study includes a limited number and range of mesenchymal tumors, not including smooth muscle tumor of uncertain malignant potential, or other types of leiomyosarcomas or leiomyomas. The current Bionano software does not include tumor purity calculation [Bionano support, personal communication (written), July 8, 2025], potentially affecting copy number calculations. However, tumor purity in the cohort was high, and distinction between homozygous and heterozygous deletions was unambiguous.

WGS and WTS require robust bioinformatics infrastructure, often involving multidisciplinary teams. In contrast, OGM is less computationally intensive and more accessible from both a cost and operational standpoint. At our institution, WGS analysis pipelines have been optimized for mesenchymal tumor profiling, including automated fusion detection. However, OGM platforms, such as Bionano Access and VIA, remain less flexible and less customizable to specific diagnostic needs. In terms of turnaround time for the analyses excluding bioinformatics, the WGS pipeline was generally faster (10 days) than OGM (30 days). The rate-limiting step in the OGM pipeline was data generation and delivery, and the turnaround time would likely decrease if it would be part of an established bioinformatic workflow. The cost for OGM was approximately a third of that of the specific WGS approach used in this study (matched tumor-normal samples and coverage 100× in tumor tissue). If sequencing would be added to the OGM approach, to capture SNVs, the cost differences would naturally decrease.

The inclusion criterion of suspected sarcoma led to the selection of relatively large, radiologically aggressive tumors, which may explain the high prevalence of genomic alterations even in histologically benign cases. Alterations in *RAD51B*, *COL4A5*, *HMGA2*, *MED12*, and *FGFR1* are consistent with previous leiomyoma studies.^33^^,^^34^ The novel *COL4A5*::*RAD51B* fusion likely represents a pathogenic event, although it has not been previously reported in leiomyomas. The *HRAS* variant in case LM-22 also lacks known associations with leiomyomas, and it could be a passenger event in this specific tumor. The substitution affects the same amino acid as a somatic hotspot for thyroid carcinoma [Catalogue of Somatic Mutations in Cancer mutation identifier COSV54239883; *https://cancer.sanger.ac.uk/cosmic*, last accessed October 20, 2025 (registration required)], and is considered likely pathogenic as a germline variant for Noonan syndrome.^14^

The *FGFR1*::*TACC1* fusion identified only by WTS in case LM-18 represents a diagnostic dilemma. Although this fusion has been linked to undifferentiated uterine sarcomas,^34^ *FGFR1* fusions have also been reported in benign leiomyomas,^35^ suggesting that the fusion partner context may influence pathogenic potential.

Finally, we report a uterine lipoblastoma harboring a *PLAG1* fusion (LB-14), which, although not described in the World Health Organization classification, aligns with the molecular features of true lipoblastoma rather than lipoblastoma-like tumors, which typically lack *PLAG1* rearrangements.^36^ Recently, a case of uterine liposarcoma with *PLAG1* rearrangement detected by fluorescence *in situ* hybridization but not in RNA analyses has been described, and the authors suggest it to be a new type of uterine liposarcoma.^37^

## Conclusion

This prospective descriptive study performed a systematic comparison of WGS and OGM for the detection of clinically relevant genomic events in gynecologic mesenchymal tumors. It shows that WGS is the most important modality in our settings, revealing SNVs, fusion events, and CNA profiles, whereas OGM gives a structural map of the genome without requiring substantial bioinformatic infrastructure. It was also found that OGM shows clear signs of being a useful alternative to conventional cytogenetic methods for mesenchymal tumor diagnostics, especially given its low demands in terms of user experience, infrastructure, and bioinformatic pipelines compared with WGS. However, when WGS is already integrated into the diagnostic workflow in the diagnostic process, the additional value of OGM is not as evident.

## Disclosure Statement

None declared.

## Supplemental Data

Supplemental Figure S1 Copy number aberrations analyzed by whole-genome sequencing (WGS) and optical genome mapping (OGM) for three leiomyosarcoma cases, visualized in a whole-genome view. BAF, B-allele frequency; LogR, log ratio; M, million; VAF, variant allele frequency.

Supplemental Figure S2 Genome instability scores compared. Comparison of fraction of genome altered (FGA) based on whole-genome sequencing (WGS) and optical genome mapping (OGM) with genomic complexity score (GCS) for each case (case LM-21 was excluded because of missing data). On the *y* axis, the scores; and on the *x* axis, each case. Malignant cases are sorted to the left, and benign to the right. Cases showing chromoanagenesis are marked by a green boxed area below (seven malignant cases, two benign cases). *n* = 24 cases. ID, identifier; LM, leiomyoma; LMS, leiomyosarcoma; PEC, perivascular epithelioid cell.
