# Supplementary material for: Optical Genome Mapping versus Whole-Genome Sequencing in the Clinical Diagnosis of Gynecologic Mesenchymal Tumors
Source: J Mol Diagn. 2025 Nov 29;28(2):187–98. doi: 10.1016/j.jmoldx.2025.11.003 (PMC12881299; doi:10.1016/j.jmoldx.2025.11.003)
Supplement: Supplemental Figure S1 — Copy number aberrations analyzed by whole-genome sequencing (WGS) and optical genome mapping (OGM) for three leiomyosarcoma cases, visualized in a whole-genome view. BAF, B-allele frequency; LogR, log ratio; M, million; VAF, variant allele frequency. [file mmc1.pdf]

Leiomyosarcoma, case LMS-12

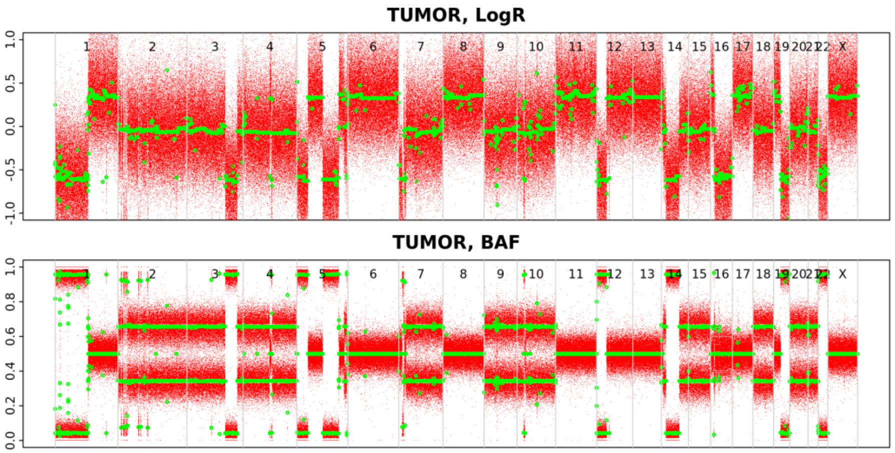

WGS

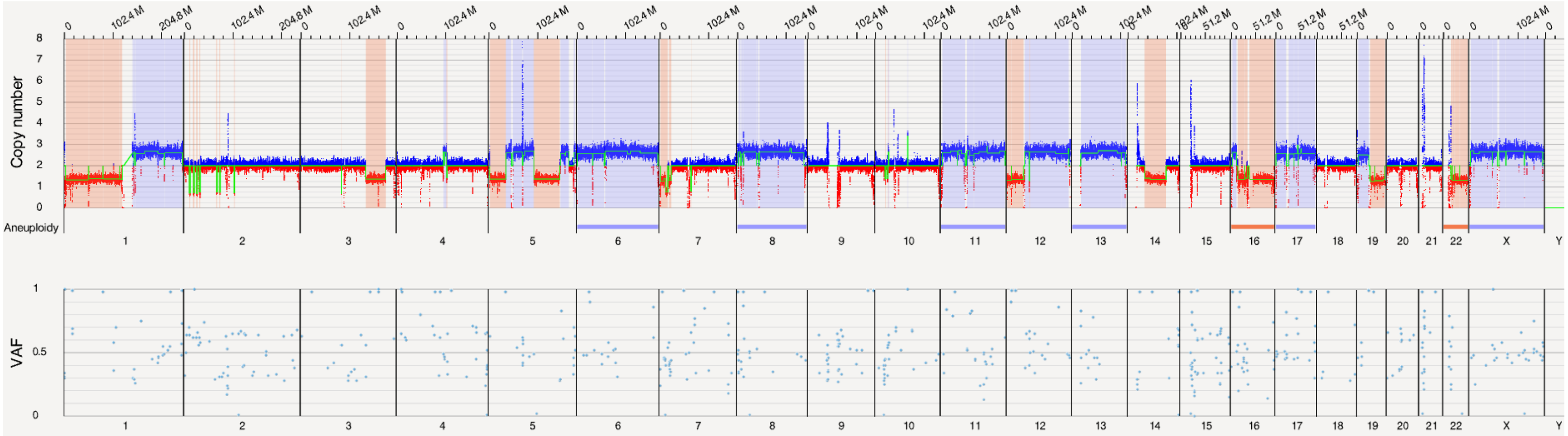

OGM

Leiomyosarcoma, case LMS-9

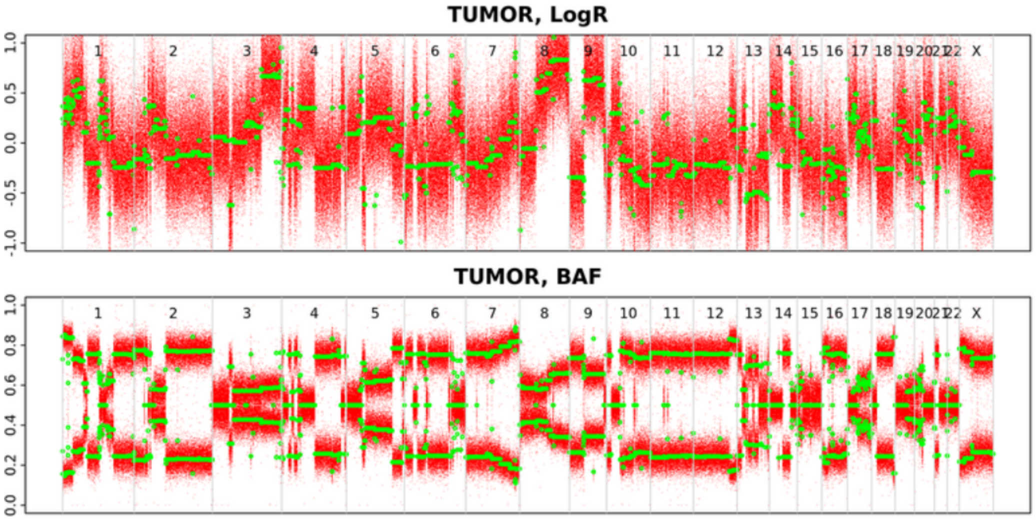

WGS

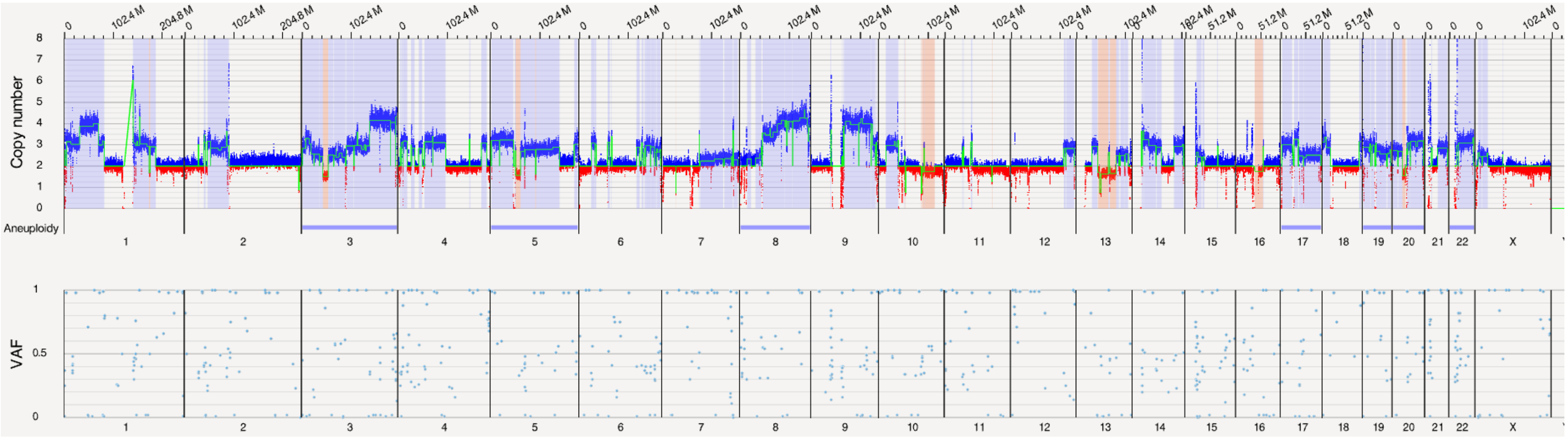

OGM

Leiomyosarcoma, case LMS-4

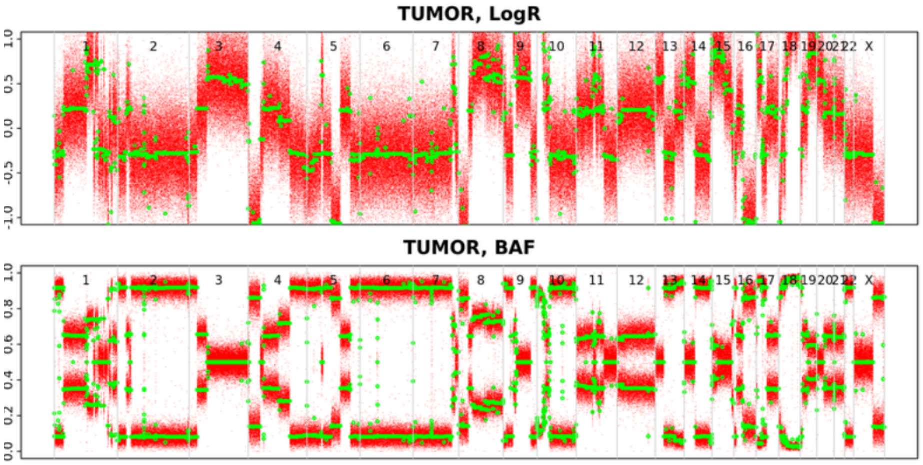

WGS

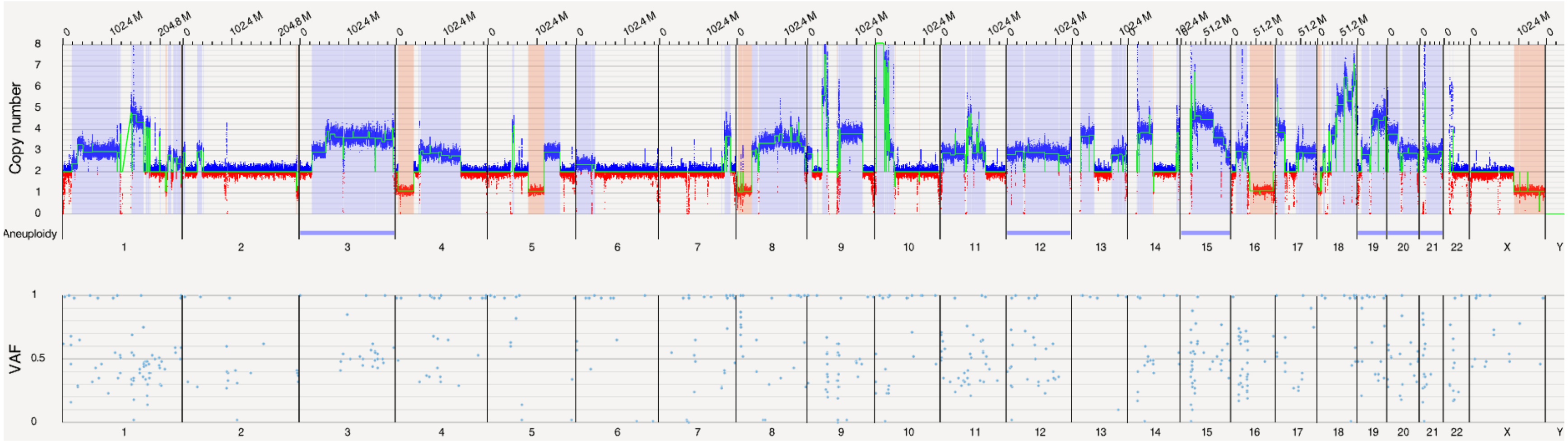

OGM
