# Supplementary figures and images for: Optical Genome Mapping versus Whole-Genome Sequencing in the Clinical Diagnosis of Gynecologic Mesenchymal Tumors
Source: J Mol Diagn. 2025 Nov 29;28(2):187–98. doi: 10.1016/j.jmoldx.2025.11.003 (PMC12881299; doi:10.1016/j.jmoldx.2025.11.003)

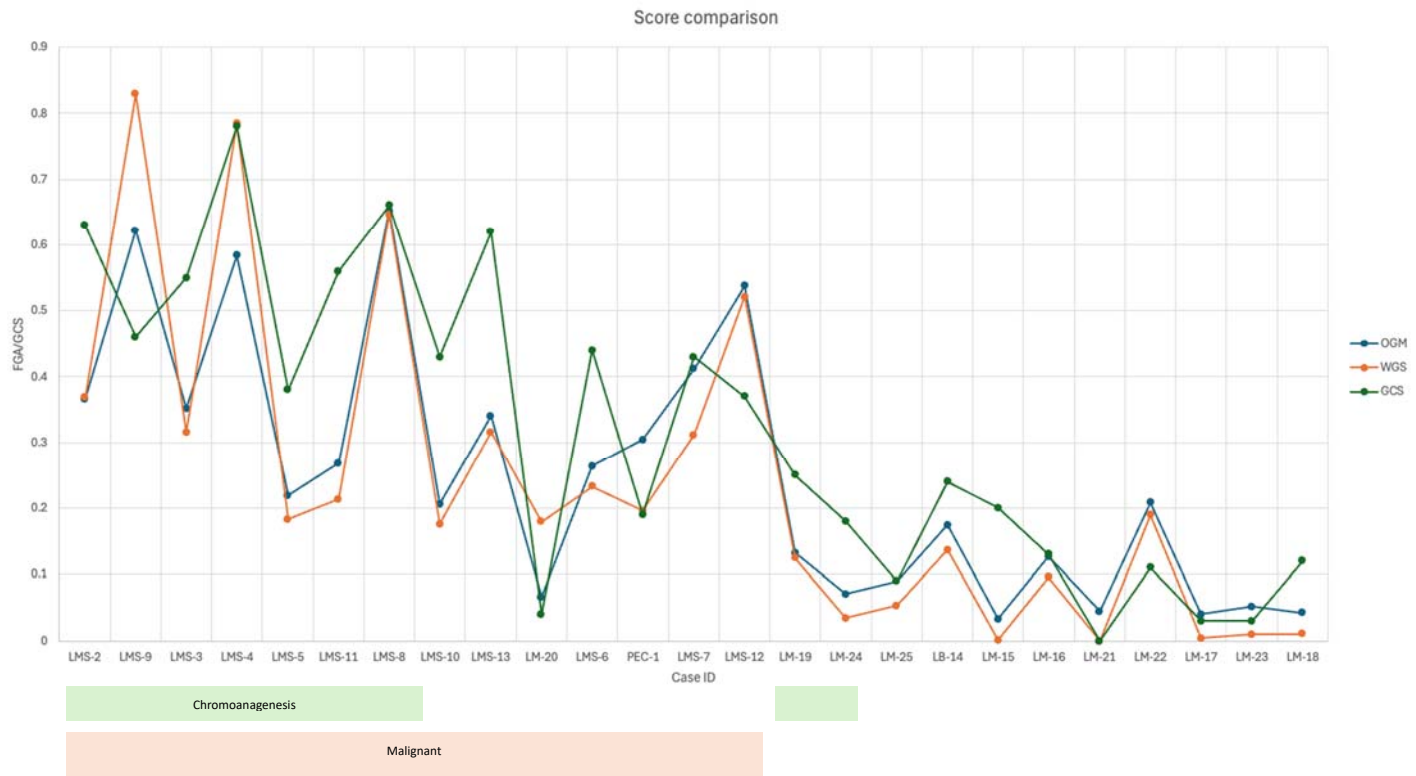

Supplement: Supplemental Figure S2 — Genome instability scores compared. Comparison of fraction of genome altered (FGA) based on whole-genome sequencing (WGS) and optical genome mapping (OGM) with genomic complexity score (GCS) for each case (case LM-21 was excluded because of missing data). On the y axis, the scores; and on the x axis, each case. Malignant cases are sorted to the left, and benign to the right. Cases showing chromoanagenesis are marked by a green boxed area below (seven malignant cases, two benign cases). n = 24 cases. ID, identifier; LM, leiomyoma; LMS, leiomyosarcoma; PEC, perivascular epithelioid cell. [file mmc2.pdf]
